# Dynamics of DNA Squeezed Inside a Nanochannel via a Sliding Gasket

**DOI:** 10.3390/polym8100352

**Published:** 2016-09-29

**Authors:** Aiqun Huang, Walter Reisner, Aniket Bhattacharya

**Affiliations:** 1 University of Central Florida, 4000 Central Florida Blvd, Orlando, FL 32816, USA; aiqunhuang@knights.ucf.edu; 2 McGill University, 845 Rue Sherbrooke O, Montréal, QC H3A 0G4, Canada; reisner@physics.mcgill.ca

**Keywords:** DNA, nanochannel, coarse-grained model, polymer physics, Brownian dynamics, nonlinear diffusion equation, statistical mechanics

## Abstract

We use Brownian dynamics (BD) simulation of a coarse-grained (CG) bead-spring model of DNA to study the nonequilibrim dynamics of a single DNA molecule confined inside a rectangular nanochannel being squeezed with a sliding gasket piston or “nanodozer”. From our simulations we extract the nonequilibrim density profile c(x,t) of the squeezed molecule along the channel axis (*x*-coordinate) and then analyze the non-equilibrium profile using a recently introduced phenomenological Nonlinear Partial Differential Equation (NPDE) model. Since the NPDE approach also fits the experimental results well and is numerically efficient to implement, the combined BD + NPDE methods can be a powerful approach to analyze details of the confined molecular dynamics. In particular, the overall excellent agreement between the two complementary sets of data provides a strategy for carrying out large scale simulation on semi-flexible biopolymers in confinement at biologically relevant length scales.

## 1. Introduction

Confined semiflexible polymers offer a variety of interesting phases and dynamics as a function of the confining length scale, such as the diameter *D* of the confining channel and the flexibility of the chain measured in terms of its persistence length $L_{p}$ [1]. For a fully flexible polymer chain it is easy to see that a quasi-one-dimensional (1D) confinement leads to a series of “blobs” [2,3] of size of the confining length *D*, each with energy ∼$k_{B}T$ [4,5,6]. In the other extreme limit when the persistence length $L_{p}\gg D$, Odijk [7] showed that it is profitable for the chain to deflect from the confining wall and meander its way through the channel with the characteristic deflection length $\lambda ∼{\left(D^{2}L_{p}\right)}^{1/3}$. More recently other intermediate phases have been introduced by Tree et al. [8,9,10] where the nature of fluctuations is different in each phase. In this paper we present results for the case where a semi-flexible polymer in a quasi-1D confinement along a narrow channel is pushed along the channel axis and thereafter allowed to relax. Evidently, the nature of the compressed state will depend on the relative ratio of channel width *D* and the chain flexibility. As a matter of fact, recently it has become possible to watch the real time non-equilibrium dynamics of a nanochannel confined DNA [11,12]. Due to recent technological breakthrough it has been possible to straighten DNA fragments of several thousand to a few millions base pairs inside nanochannels [13,14,15,16,17,18] and characteristic patches in a sequence consisting of several thousand base pairs called “barcodes” [19,20] have been determined from different melting probabilities of AT (Adenine-Thymine) and GC (Guanine-Cytosine) bonds. Equilibrium DNA conformations have also been studied inside a tapered nanochannel so as to study the effect of confinement in a continuous fashion [21]. Equilibrium results for confined DNA have been quantified using scaling concepts of polymer physics [2], supplemented by a large number of simulation studies both in three dimensions (3D) [8,9,10], and in two dimensions [22,23,24]. Compared to the well-established equilibrium results, non-equilibrium dynamics of confined DNA are poorly understood, with only a few studies [11,12,25,26]. The earlier experiment of Reccius et al. [25] studied the sudden expansion of a confined DNA strand by turning off the electric field and watched the expansion of the strand in real time. Reccius et al. used the expression of equilibrium free energy of a confined flexible polymer [4,5] generalized for a semi-flexible chain by Schaefer et al. [27] to explain their findings [6]. Pelletier et al. observed the real time force compression dynamics of bacterial chromosome by using a “micro-piston” [26]. More recently, Khorshid et al. [11,12] studied dynamics of compression and subsequent relaxation of a squeezed DNA inside a 1D nanochannel using an optically trapped nanosphere (a “nano-dozer”). In another experiment, the same group has shown using the same compression technique that it is possible to observe knot formation along the DNA and the subsequent unraveling of the knots at the molecule free ends [28]. The evolving density profile c(x,t) of such a process has been obtained from phenomenological nonlinear partial different equation (NPDE) based approach [12]. Compared to Brownian dynamics (BD) simulations on coarse-grained (CG) models of polymers, the NPDE approach is very fast, and thus can be used as a supplementary tool for analyzing experimental results for confined DNA and other biopolymers. On the other hand, unlike BD, it does not provide details of the subchain conformations and dynamics. Thus a combination of these two complementary methods (NPDE + BD) can be quite powerful to unravel dynamics of DNA and other long biomolecules. In this paper, we explore the advantage of this hybrid multiscale method. The organization of the rest of the paper is as follows. In the next section we provide some details of the BD simulation and the NPDE approach. In Section 3 we present the data for the BD simulation and fit these data with NPDE formalism. We provide a summary and an outlook of this approach for future work in Section 4.

## 2. DNA Simulation Approaches

In this section we introduce two approaches for studying the dynamics of DNA squeezed inside a nanochannel. In the first approach, we use a coarse-grained (CG) bead-spring model of DNA incorporating semiflexibilty and excluded-volume. This model retains the key features for modelling DNA polymer conformation at scales greater than the chain persistence length. Our second approach is based on a phenomenological NPDE formalism, where the initial conditions and the boundary conditions respect the chain connectivity. The NPDE approach yields the time-evolution of the density profile c(x,t) along the channel axis (*x*-axis). The density profile from the NPDE formalism is then fit to the density profile obtained from the BD simulation. The NPDE approach is computationally less demanding than the bead-spring model. In particular, by suitably interpreting the CG parameters of the BD simulation model, the NPDE approach can be used to bridge the length and time scales between BD simulations and the experimental data. A combination of the two approaches can be enormously profitable to study DNA dynamics at various length and time scales.

### 2.1. Bead-Spring Model and BD Simulation

**Figure 1:**
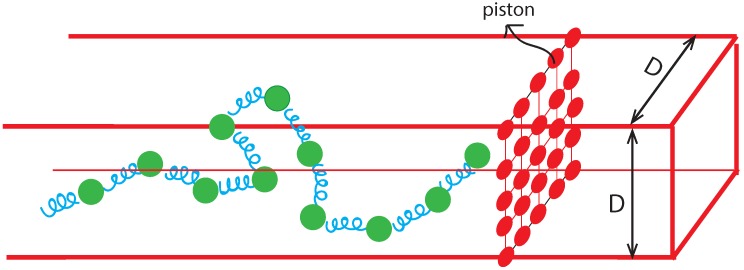
A bead-spring model of semi-flexible chain confined inside a nanochannel of width *D* being pushed by a sliding gasket piston (nano-dozer). The green beads are connected via anharmonic spring potentials and interact at long-range via excluded-volume. The red wall particles are at a fixed distance from each other on a square lattice and move together at the piston velocity. The chain always remains confined inside the channel.

We have used a “bead-spring” model of a polymer chain (Figure 1) originally introduced for a fully flexible chain by Grest and Kremer [29]. The model has been studied quite extensively by many groups using both Monte Carlo (MC) and various molecular dynamics (MD) methods. Recently we have generalized the model for a semi-flexible chain and studied both equilibrium and dynamic properties [30]. Comparison of our BD results with those obtained for very large self-avoiding chains on a square lattice reveals robustness of the model for certain universal aspects, e.g., scaling of end-to-end distance and transverse fluctuations [22,23,24,30], providing more confidence in using this model to study nonequilibrim dynamics of confined polymers. Here, we will also observe that the overall agreement of the results from the BD approach with those obtained from the NPDE approach is remarkable, which we believe will enable us to compare results for very long length and time scales.

Figure 1 shows the CG model of the system that we used to carry out BD simulation. A double-stranded DNA (dsDNA) (experiments are carried out with *λ*-phage or T4 [11,12]) is modelled as a bead-spring polymer chain. The diameter of each bead $\sigma_{b}$ (Equation (1)) is an adjustable parameter to be interpreted to compare results for a specific system. The Hamiltonian incorporates an excluded volume (EV) interaction, an anharmonic spring bond potential and a bending potential (Equations (1)–(3) respectively).

Specifically, the EV effect, acting between any two beads along the chain, is implemented as a short range purely repulsive Lennard-Jones (LJ) potential (Equation (1)).

(1) $$\begin{array}{ccc} U_{\mathrm{LJ}}\left(r_{b}\right) & = & 4\epsilon \left[{\left(\frac{\sigma_{b}}{r_{b}}\right)}^{12}-{\left(\frac{\sigma_{b}}{r_{b}}\right)}^{6}\right]+\epsilon \;\;\;\;\;\;\;\;\mathrm{for}\;\;r_{b}\leq 2^{1/6}\sigma_{b}\\ & = & 0\;\;\;\;\;\;\;\;\;\;\;\;\;\;\;\;\;\;\;\;\;\;\;\;\;\;\;\mathrm{for}\;\;r_{b}>2^{1/6}\sigma_{b}\;, \end{array}$$

where *ϵ* is the strength of the potential, $\sigma_{b}$ is the diameter (size) of the beads and $r_{b}$ is the distance for any pair of beads. The LJ potential is nonzero only within a cutoff range of $2^{1/6}\sigma_{b}$. The confining walls consist of equally spaced immobile LJ particles of the same diameter $\sigma_{b}$. The EV interaction between the polymer beads and the confining wall particles are given by Equation (1). The piston is modelled as beads of diameter $\sigma_{b}$ held at fixed separation on a square lattice. The purely repulsive DNA-piston interaction is also described by Equation (1). Constructing the piston this way ensures that the piston is impermeable to polymer particles.

The bond potential acting between any two successive monomers (beads) along the chain is modelled as a finitely extensible nonlinear elastic (FENE) interaction [29]

(2) $$U_{\mathrm{FENE}}\left(r_{m}\right)=-\frac{1}{2}kR_{0}^{2}\ln\left(1-r_{m}^{2}/R_{0}^{2}\right),$$

where *k* is the spring constant, $r_{m}$ is the distance between two consecutive monomers, $R_{0}$ is the allowed maximum bond length. The bending energy of the chain is described by an angle dependent potential

(3) $$U_{\mathrm{bend}}=\kappa \left(1-\cos\theta \right),$$

where *θ* is the angle made by two successive bonds and *κ* is the controlling parameter for the rigidity of the chain. In three dimensions, for κ≠0, the persistence length $L_{p}$ of the chain is related to *κ* via [31]

(4) $$L_{p}=\frac{\kappa }{k_{B}T},$$

where $k_{B}$ is the Boltzmann constant and *T* is the temperature. While this relation is strictly valid for a worm like chain [3] without the EV interaction, we have verified from BD simulation on the same bead-spring model of a semi-flexible chain that Equation (4) remains valid in presence of the EV interaction (this is necessarily so, as the chain persistence length is a local phenomenon) and even for low values of *κ* [23]. For the results reported in this paper Equation (4) is well obeyed. We use the following Langevin dynamics equation of motion to advance the position of the *i*th monomer

(5) $$m{\ddot{r}}_{i}=-\nabla \left(U_{\mathrm{LJ}}+U_{\mathrm{FENE}}+U_{\mathrm{bend}}+U_{\mathrm{wall}}+U_{\mathrm{piston}}\right)-\gamma v_{i}+R_{i},$$

where *γ* is the monomer friction coefficient, and $R_{i}$ is a Gaussian random force with zero mean at temperature *T*, and satisfies the fluctuation-dissipation relation $\langle R_{i}\left(t\right)\cdot R_{j}\left(t^{\prime }\right)\rangle =2Ddk_{B}T\gamma \;\delta_{ij}\;\delta \left(t-t^{\prime }\right)$ in *d* dimensions. Here $k_{B}$, *D* and $\delta_{ij}$ are the Boltzmann constant, the diffusion constant and the Kronecker delta ($\delta_{ij}=0$ for i≠j and $\delta_{ij}=1$ for i=j) respectively. The numerical integration of Equation (5) is implemented using the algorithm introduced by Gunsteren and Berendsen [32]. Length and energy units are respectively $\sigma_{b}$ and *ϵ*; *m* represents the mass of the order of a nucleotide. Consequently, the reduced units for length, time and the temperature are *σ*, $\sigma \sqrt{\frac{m}{\epsilon }}$, $\epsilon /k_{B}$ respectively. Our previous experience with BD simulation suggests that appropriate parameter specifications are γ=0.7, k=30, $R_{0}=1.5$, and the temperature T=1.2. These parameters values are stable for a long time and do not lead to unphysical crossing of a bond by a monomer [23,24]. The average bond length stabilizes at $b_{l}=0.97\pm 0.002$ with negligible fluctuation regardless of the chain size and rigidity [23,33]. We further assume that the electrostatic interactions are heavily screened and the EV, bond stretching and bond bending potentials (Equations (1)–(3)) are adequate to represent the physical system.

### 2.2. The Nonlinear Partial Differential Equation (NPDE) Approach

Here we briefly discuss the relevant points of this approach for comparison to our BD simulation results. More details can be found in our previous publications [11,12]. The concentration profile *c* of the confined chain (hereafter concentration *c* is defined as the number density of the beads) can be modeled via the diffusion equation:

(6) $$\frac{\partial c}{\partial t}-\frac{\partial }{\partial x}\left(D_{c}\left(c\right)\frac{\partial c}{\partial x}-cV\right)=0.$$

Here $D_{c}\left(c\right)$ is the cooperative diffusion constant, itself a function of the local polymer concentration. The quantity *V* is the compressional velocity, which we identify with $V_{\mathrm{push}}$. By analyzing the structure of the steady-state ramp obtained from experiments, we can, —on a purely phenomenological basis, —find $D_{c}$ as a function of *c*. In particular, the linear structure of the steady-state profile implies that $D_{c}$ is linearly proportional to *c*, a relation we parameterize via $D_{c}=D_{o}\left(c/c_{o}\right)$ with $c_{o}$ the equilibrium concentration of the extended chain and $D_{o}$ a constant that can be extracted from knowledge of the steady-state ramp slope (e.g., Figure 3f).

Equation (6), taken in isolation, would predict that an initially localized concentration distribution c(x,t) would decay to zero in the limit of large times. Of course, in the context of a single polymer, such behavior is unphysical: chain connectivity implies that the concentration distribution must remain bounded and finite. A compressed molecule will relax to reach a bounded concentration distribution with the character of a uniform plateau with constant concentration $c_{o}$. To build in this requirement, we supplement Equation (6) by two other equations, applied to the chain ends, that halt the relaxation process when $c=c_{o}$. For the purposes of this model, we assume that the concentration at the chain edges falls off over a scale that is small in comparison with $r_{o}$ and on order of the blob size [2,6,8,9] (in our case $r_{0}∼D$). The first equation determines how external forces at the chain edges, including osmotic pressure and the frictional force due to the sliding speed, induce edge motion:

(7) $$\xi_{i}\frac{dx_{i}}{dt}=\mp \Pi D^{2}+\xi_{i}V.$$

Here the quantity Π is the osmotic pressure at the chain edges, also a function of $c(x_{i},t)$. The subscripts i=1 and 2 refer to the left and right chain edges respectively. Likewise, $\xi_{i}$’s are the friction factors for the chain edges. The remaining equation is the flux balance equation for the chain edges and is given by

(8) $$c\left(x_{i},t\right)\frac{dx_{i}}{dt}=-{\left.D_{c}\frac{\partial c}{\partial x}\right|}_{x=x_{c}}+c\left(x_{i},t\right)V.$$

Equations (6)–(8) can be solved self consistently to obtain c(x,t). A crucial input for solving Equations (6)–(8) that was used by Khorshid et al. [12] is an expression for the free energy per chain for the extended de Gennes regime

(9) $$\frac{f}{k_{B}T}=\frac{A}{r_{b}}\left[-\frac{r}{r_{0}}+{\left(\frac{r_{0}}{r}\right)}^{2}\right],$$

from which the dependence of the osmotic pressure Π can be obtained in terms of c(x,t).

(10) $$\Pi =\Pi_{0}\left[-\frac{c_{0}}{c}+{\left(\frac{c}{c_{0}}\right)}^{2}\right].$$

We use the same set of equations to fit the BD simulation data as shown in Figure 3, Figure 4, Figure 5 and Figure 6. Note that the concentration and extension of the equilibrium chain ($c_{o}$ and $r_{o}$) can be used to normalize concentration and length scales in the problem; all results are shown in terms of reduced concentration and position variables $c/c_{o}$ and $x/r_{o}$. Moreover, the constant $D_{o}$, with units of diffusivity, introduces a time-scale $\tau =r_{o}^{2}/D_{o}$ which can be used to introduce a velocity scale $V_{o}=r_{o}/\tau$. The steady-state ramp-slope for a profile in reduced concentration and position variables has the value $V/V_{o}$ so that $D_{o}=\left(V/V_{o}\right)r_{o}$ (see [12]). In order to apply the NPDE model, we adopt exactly the same procedure used to analyze the experimental data in [12]. We first obtain $D_{o}$ from the slope of the linear ramp in the steady state profile. We then solve the NPDE model for an initial concentration profile c(x,t=0) appropriate to the problem. For the compression problem, c(x,0) is a uniform equilibrium profile with concentration $c_{o}$. For the retraction problem, c(x,0) is the steady-state ramp-profile created by the initial chain compression. However, there is still a missing parameter: the edge friction $\xi_{i}$. In the NPDE model this is a purely phenomenological parameter that must be obtained from least-squares fitting. When applying the NPDE approach to profiles expressed in reduced variables, we extract the dimensionless parameter $\alpha =\xi D_{o}/\Pi_{0}D^{2}r_{o}$.

Lastly, when fitting the NPDE results to the BD output, we must deal with an extremely subtle issue: the presence of thermal fluctuations of the molecule extension that have the effect of broadening the profile edges. We choose, for the purposes of this preliminary study, to argue that the extensional fluctuations can be described by convolving the NPDE output with a Gaussian function: $G\left(x-y\right)=\left(1/\sqrt{2\pi \sigma }\right)\exp(-{\left(x-y\right)}^{2}/2\sigma^{2})$ with *σ* the “width” of the convolution function. To explain on a heuristic basis why this might be reasonable, imagine a population of particles in a harmonic trapping potential. If no thermal fluctuations are present, the particles are all trapped at the potential equilibrium position, e.g., constituting a delta-function concentration profile. If fluctuations are present, the delta-function is smeared into a Gaussian shape with a width *σ* given by the rms excursion of a particle in the well. To a very crude first approximation, we can imagine our coarse-grained polymer as a superposition of harmonic trapping sites: the polymer segments at a given position and at short-times are harmonically bound and fluctuate about the equilibrium position of the confinement potential (e.g., given by Equation (9)). If we assume that each trapping site varies slowly enough, e.g., on times scales slow compared to the relaxation time in the trap, then we argue that the concentration profile on long time-scales can be assumed to evolve through the NPDE equations with the short-time fluctuations handled by the Gaussian convolution. Note that even in this approximation, we might expect the width of our convolution function–corresponding to the rms excursion of a polymer segment at a given position–to depend on the local concentration. We choose to ignore this complication and assume that *σ* is constant. In particular, we obtain *σ* from fits to the initial profile. For the compression results, we do allow for the edge of the molecule closest to the gasket to have less thermal broadening (e.g., we assume distinct *σ*-values for edge near gasket and the edge opposite the gasket); for the retraction results we assume that both edges have the same degree of broadening.

## 3. Results

**Figure 2:**
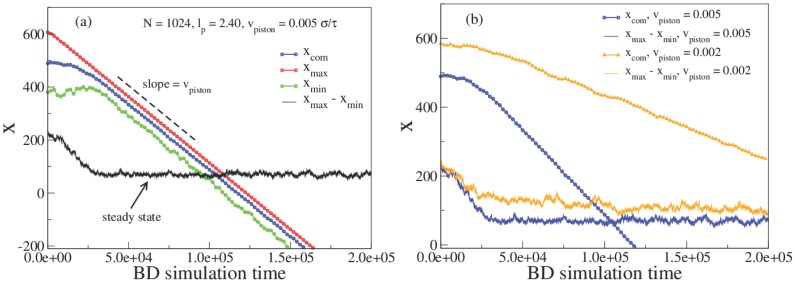
(**a**) The evolution of the maximum (green), minimum (red) and the *x*-coordinate of the center of mass (COM) (black) (along the channel axis) during the simulation are shown, the extension of the chain The piston is approached from the $x_{\max}$ side. When the COM’s speed becomes the same as $V_{\mathrm{push}}$, the steady state of the polymer chain is achieved; (**b**) The same as in (**a**) but compares the effect of two different piston speeds 0.005 and 0.002 respectively.

We now show the BD simulation results along with the those obtained from the NPDE approach. In setting up our BD simulation we closely followed Ref. [11,12] to mimic the actual experiment. All of our simulations are carried out for a chain of length N=1024 confined in a rectangular nanochannel of width D=16. For most of the results we have set the chain flexibility parameter κ=3.2 (or persistence length, $L_{p}=\kappa /k_{B}T=2.4$); however, for a few cases we have studied the effect of increasing the chain persistence length. However, for all of our cases studied here, the ratio $L_{p}/D\leq 1.0$, so that the equilibrium configurations are either in the de Gennes or in the extended de Gennes regime [8,9]. The coarse-grained chain concentration is obtained from summing the number of bond lengths in bins of size 2 and then normalizing to the total chain length. The profiles are further averaged over an ensemble of 10 independent runs. We measure time in units of “snapshots” (=1000 simulation iterations) or MD time units (=100 iterations or 0.1 snapshots). As in the experiment of Ref. [12], the piston approaches the polymer chain (right end in Figure 1) at a sliding speed of $V_{\mathrm{push}}=0.05$ (units of bond length per snapshot), and then retracts at a speed of $V_{\mathrm{retract}}=4V_{\mathrm{push}}$. A first look at the simulation results is shown in Figure 2.

**Figure 3:**
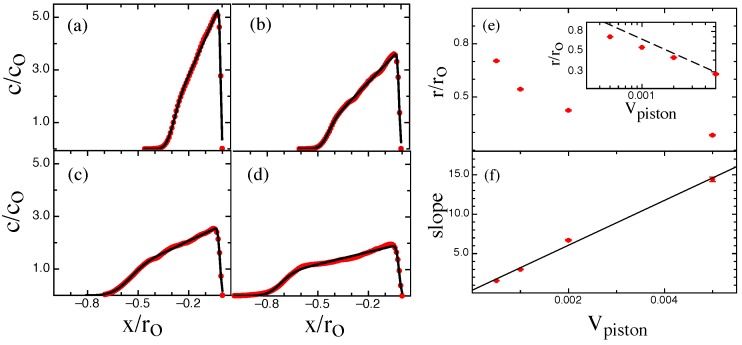
The steady state chain density c(x,t) along the channel axis for (**a**) a piston velocity $V_{\mathrm{push}}=0.05$; (**b**) $V_{\mathrm{push}}=0.02$; (**c**) $V_{\mathrm{push}}=0.01$ and (**d**) $V_{\mathrm{push}}=0.005$. (units of bond length per snapshot). The red lines are Brownian dynamics (BD) simulation output and the black lines represent the linear ramp steady-state profile predicted by the Nonlinear Partial Differential Equation (NPDE) model. Note that the linear ramp is convolved with a Gaussian function to represent the effect of thermal fluctuations that create an effective broadening of the profile shape. The extracted extension and ramp-slopes from the NPDE model fits (**a**–**d**) are shown in (**e**,**f**). The inset to (**e**) shows the same data on a log-log scale against a power law $∼V_{\mathrm{push}}^{-0.5}$ (dashed-line). The bold line in (**f**) is a best linear fit showing that the extracted slopes are linearly proportional to the pushing speed.

We plot the *x*-coordinate of the chain center of mass (COM), $X_{\mathrm{COM}}$, the chain left and right edge ($x_{\min}$ and $x_{\max}$ respectively) and the chain extension $r=x_{\max}-x_{\min}$ during the compression process. The chain is confined in a rectangular nanochannel that for all practical purposes extends to infinity along the negative *x*-axis. The channel geometry’s translational symmetry ensures that the molecule is free to move in the direction of the velocity of the nano-dozer along the *x*-axis and that the chain sees the same confinement when displaced horizontally. For our model parameters the equilibrium extension of the chain along the channel $r_{o}=235$ (in units of bond-length $b_{l}$). At the very outset of the compression process, the molecular contour will build up locally at the chain edge adjacent to the moving nanodozer. The chain will then undergo transient compression with the chain dynamically evolving from a uniform equilibrium to a ramped steady-state profile. Once the steady-state is reached, as the chain is free to slide down the channel, the entire chain will translate at the piston speed while the profile shape maintains the ramped steady-state structure. In particular, as shown in Figure 2a, after an initial transient phase the respective slopes of $X_{\mathrm{COM}}$, $x_{\min}$, and $x_{\max}$ will become equal and the chain extension $X=x_{\max}-x_{\min}$ will approach a constant steady-state value. At higher speed the chain will become more compressed and the steady state chain extension is reduced. The steady-state chain density is plotted in Figure 3 for different piston speeds and compared to the predicted steady-state profile for the NPDE approach. The NPDE steady-state profile is simply a linear ramp, in accord with experimental observations [11]. Note that the presence of thermal fluctuations leads to an effective “broadening” of the chain profile. We treat this broadening in a purely descriptive fashion by applying a Gaussian convolution to the NPDE model-output with a width determined by fitting to the chain initial profile. The extension versus sliding speed, shown in Figure 3e, appears to asymptote to the power law $r∼V_{\mathrm{push}}^{-0.5}$, suggesting that these simulations results were obtained for a sliding speed on order of the critical speed identified in [11]. In addition, we find that the ramp slope is linearly proportional to the sliding speed (Figure 3f, again in agreement with [11]).

**Figure 4:**
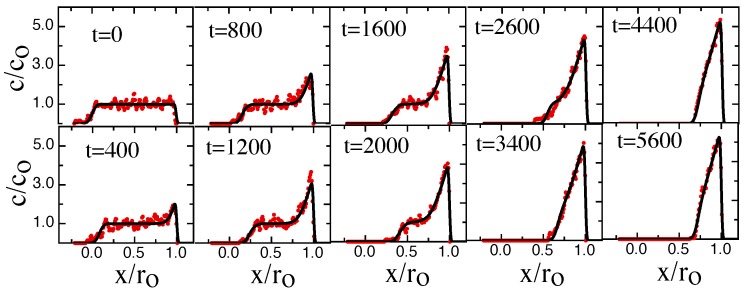
Time evolution of the normalized chain density $c\left(x,t\right)/c_{0}$ along the channel axis for various times during compression with a sliding gasket moving at speed $V_{\mathrm{push}}=0.05$. The channel width D=16 and the persistence length $L_{p}=2.5$. Time-values are shown in snapshot units, with one snapshot corresponding to 1000 iterations. The red circles correspond to the BD simulation data; the black solid lines correspond to the fitted prediction from the NPDE model. The NPDE model is solved with a $D_{o}=0.74$ (bond unit squared per snapshot, obtained from the ramp-slope in steady-state); this $D_{o}$ value leads to a τ=75,000 (snap-shot units). The best-fit β=0.07. The *σ* values were determined from a fit of a uniform concentration profile model to the profile at t=0. For the edge near the gasket, $\sigma =0.008r_{o}$. For the edge opposite the gasket, $\sigma =0.05r_{o}$.

Next we focus on the non-equilibrium configurations in the transient phase during the approach to steady state. Figure 4 shows a time-series of chain concentration profiles c(x,t) when the chain is dynamically compressed.

**Figure 5:**
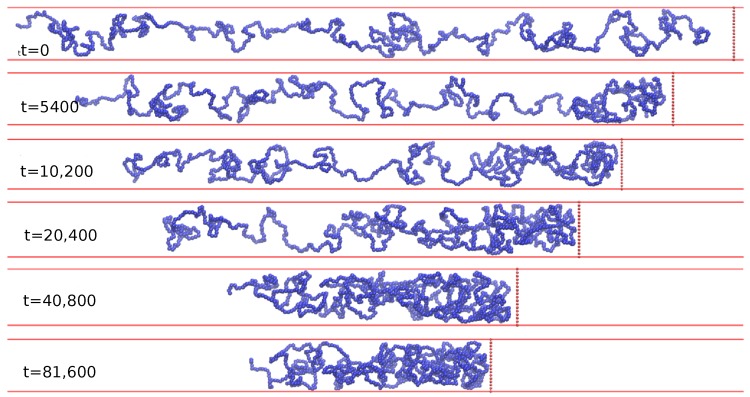
The corresponding snapshots of the simulated chain configuration for different c(x,t) in Figure 4.

**Figure 6:**
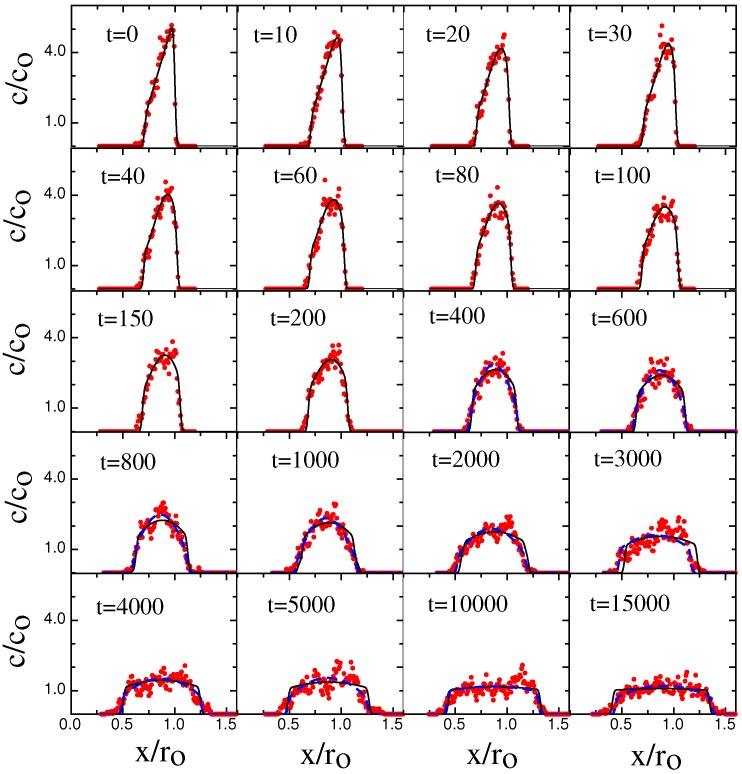
Time evolution of the normalized chain density $c\left(x,t\right)/c_{0}$ along the channel axis during bead retraction. The channel width D=16 and the persistence length $L_{p}=2.5$. The chain profile is compressed by moving the gasket at a sliding speed $V_{\mathrm{push}}=0.05$; the bead is then retracted at speed $V_{\mathrm{retract}}=4V_{\mathrm{push}}$, inducing relaxation of the profile. Time-values are shown in snapshot units, with one snapshot corresponding to 1000 iterations. The red circles correspond to the BD simulation data; the black solid lines correspond to the fitted prediction from the NPDE model. The dashed blue curves for t>400 correspond to fits to a parabolic concentration model. The NPDE model is solved with a $D_{o}=0.74$ (obtained from the ramp-slope in steady-state); this $D_{o}$ value leads to a τ= 75,000. The best-fit β=0.26. The value of $\sigma =0.013r_{o}$, determined from a fit of a broadened ramp profile model to the profile at t=0.

Typical chain configuration snapshots for these times are shown in Figure 5 when the chain density progressively increases. When the steady state is achieved (as evident from the plateau in Figure 2) then the chain is allowed to retract. Figure 6 shows a time-series of chain concentration profiles c(x,t) for this retraction phase.

Please note that both Figure 4 and Figure 6 BD-results (red circles) are compared to NPDE model output (solid black lines).

Note that our BD results have a qualitative character very similar to the experimental results in [12]. At t=0 right before the piston starts to slide, the chain density shows a uniform plateau along the channel axis. When the gasket contacts the molecule, the transient compression process begins with a “shockwave” of locally enhanced concentration growing near the piston edge and then propagating down the chain. The concentration profile in this regime is a combination of the unaffected plateau and the shock-wave. When the shockwave reaches the chain edge opposite the sliding gasket, the plateau disappears and the profile slowly evolves towards its steady-sate configuration, with the edge concentration gradually rising to its steady-state value.

The relaxation process begins with a fast evolution of the initial ramped concentration profile (t<400). This evolution gradually drives the concentration profile into an approximately parabolic profile shape for intermediate times (400<t<5000). At longer times, the profile shape approaches a flat equilibrium profile at the equilibrium concentration $c_{o}$. In order to remove the effects of center-of-mass diffusion from the retraction profiles, which would lead to additional broadening of the profiles, the profiles at different times have been shifted so that the position of the center-of-mass remains constant. The agreement between the BD-results and NPDE model is excellent but not perfect. The NPDE model captures the BD results extremely well on a semi-quantitative basis, for example predicting the evolution of the shock-wave and the gradual approach towards the steady-state profile (Figure 4). There are details, however, that the NPDE approach does not capture, for example the concentration tail at t=2600 in Figure 4 is not described correctly.

**Figure 7:**
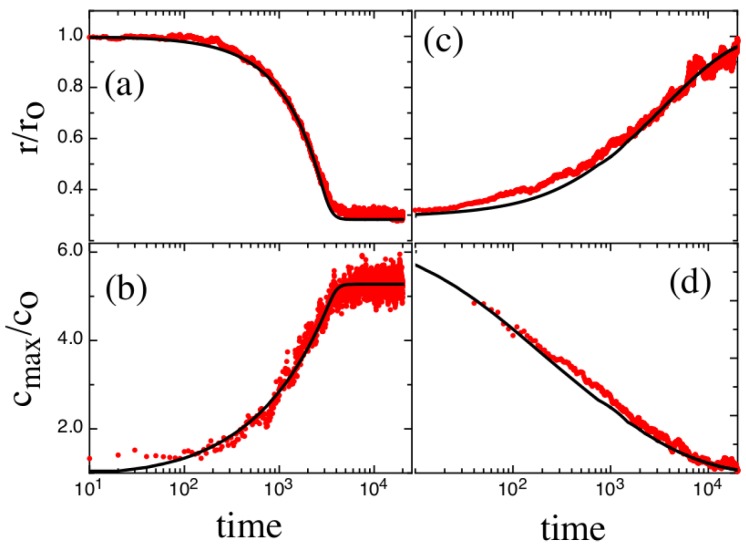
The chain extension and maximum concentration for a dynamic compression (**a**,**b**) and retraction (relaxation) process (**c**,**d**). The red line corresponds to BD results; the black curve corresponds to NPDE model output corresponding to the profile fits shown in Figure 4 and Figure 6. The maximum profile concentration for the BD output for the transient compression process is obtained by taking a three-point running average of the simulation data to suppress concentration fluctuations at the peak position. The maximum profile concentration for the BD output for the retraction process is obtained by performing a fit of the concentration profile to a parabolic concentration model. The approach minimizes the influence of thermal fluctuations on the concentration maximum as the profile approaches equilibrium.

This is also seen in Figure 7 which shows the BD chain extension and profile maximum concentration $c_{\max}$ versus time for the profiles in Figure 4 and Figure 6. We note that the NPDE-approach slightly underestimates the correct steady-state extension-value (Figure 7a). These issues probably arise from the crude way that thermal fluctuations are handled. The relaxation process (Figure 6) is more difficult for the NPDE approach to capture. While the short-time behavior is described extremely accurately (t<200), the intermediate profiles are not described as well. The BD-results do have an approximately parabolic character, as predicted in [12], but the NPDE model underestimates the chain maximum. Note that to emphasize the parabolic character of the retraction profiles, and also obtain a better measure of the maximum concentration (e.g., that averages over thermal fluctuations present in the profiles).

**Figure 8:**
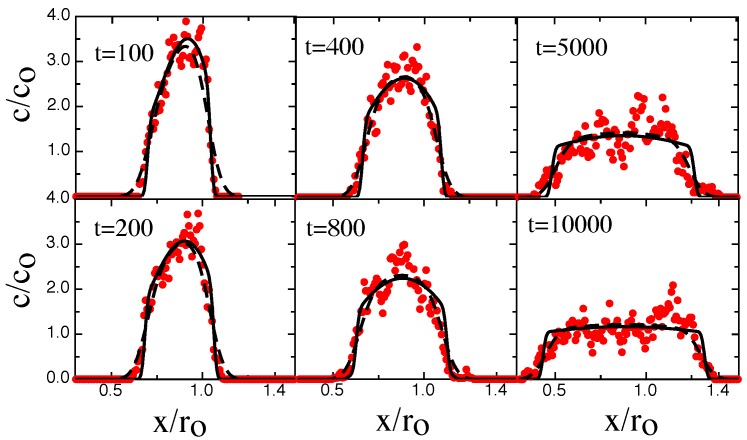
The red circles are results of coarse-graining BD-simulations for the retraction profile. The bold black curve is the NPDE model output with the degree of extensional fluctuations determined from the initial profile ($\sigma =0.013r_{o}$); the dashed bold curve is the NPDE model output with the degree of extensional fluctuations determined by fits to the equilibrium profile ($\sigma =0.06r_{o}$). The degree of thermal broadening clearly increases at longer times as the profile approaches equilibrium.

We have fitted the retraction profiles to a parabolic concentration model convolved with a Gaussian (the extracted parabola amplitude is what is shown in Figure 7d). At longer times, we also see that the BD-profiles are broader than that predicted by the NPDE approach: this effect probably arises as the assumption of a fixed degree of extensional fluctuations is too crude. Extensional fluctuations become stronger at lower concentration, leading to an increased smearing of the profiles. One alternative is to fix *σ* using the degree of broadening present at very long times; naturally, this approach leads to an overestimate at short-times (see Figure 8).

## 4. Summary and Outlook

In summary, motivated by recent experimental results, we have studied the steady state as well as the nonequilibrim dynamics of compression and relaxation of a dsDNA confined and squeezed inside a nanochannel using BD simulation with a bead-spring model of polymer. We find that a NPDE approach recently introduced by Khorshid et al. [12] fits the BD simulation data very well. The cases reported here are for $L_{p}/D\leq 1$ so that the chain persistence length is small compared to the channel width, and the equilibrium phases belong to either de Gennes or extended de Gennes regimes. Here we find that the NPDE approach is a very powerful phenomenological approach to fit the experimental as well as the simulation data. However, for $L_{p}/D\geq 1$ the equilibrium configurations will correspond to the Odijk [7] deflections segments from the wall. Thus one can imagine that the squeezed states of these stiff polymers can be very different than those reported here. A different free energy expression than the one used by Khorshid et al. [12] (Equation (9) in this paper) might be necessary for ready convergence of the NPDE equations. Whatever they are, the physical origin of the parameters in the NPDE equations (e.g., *A* and $r_{b}$ in Equation (9)) can not be deciphered. The evolving configurations from the BD simulation can be immensely useful in constructing an approximate free energy landscape to start and in principle will provide all the details of the time dependence from the CG bead level to collective conformation and dynamics of the entire chain. In this paper we have provided some results of these comparisons to demonstrate the success of the approach. In future we would like to explore nonequilibrim dynamics of stiffer chains using a larger parameter space, clarify the edge friction by varying molecule length and sliding speed and explore approaches to more effectively capture the time and concentration dependent thermal broadening present in the profiles.
